# A Survey of Authentication in Internet of Things-Enabled Healthcare Systems

**DOI:** 10.3390/s22239089

**Published:** 2022-11-23

**Authors:** Mudassar Ali Khan, Ikram Ud Din, Tha’er Majali, Byung-Seo Kim

**Affiliations:** 1 Department of Information Technology, The University of Haripur, Haripur 22620, Pakistan; 2 Department of Management Information Systems, Applied Science Private University, Shafa Badran, Amman 11937, Jordan; 3 Department of Software and Communications Engineering, Hongik University, Sejong 30016, Republic of Korea

**Keywords:** authentication, healthcare, Internet of things, IoMT, security, vulnerability

## Abstract

The Internet of medical things (IoMT) provides an ecosystem in which to connect humans, devices, sensors, and systems and improve healthcare services through modern technologies. The IoMT has been around for quite some time, and many architectures/systems have been proposed to exploit its true potential. Healthcare through the Internet of things (IoT) is envisioned to be efficient, accessible, and secure in all possible ways. Even though the personalized health service through IoT is not limited to time or location, many associated challenges have emerged at an exponential pace. With the rapid shift toward IoT-enabled healthcare systems, there is an extensive need to examine possible threats and propose countermeasures. Authentication is one of the key processes in a system’s security, where an individual, device, or another system is validated for its identity. This survey explores authentication techniques proposed for IoT-enabled healthcare systems. The exploration of the literature is categorized with respect to the technology deployment region, as in cloud, fog, and edge. A taxonomy of attacks, comprehensive analysis, and comparison of existing authentication techniques opens up possible future directions and paves the road ahead.

## 1. Introduction

The Internet of medical things (IoMT) allows a variety of medical equipment and applications to communicate over the Internet, which has redefined healthcare over the past few decades [1,2]. Throughout the medical sector, wearable Internet of things (IoT) technologies have unleashed the era of smart healthcare [3,4], enabling the constant monitoring of patients under safe living conditions and thereby strengthening the existing hospital facilities [5,6,7].

According to the World Health Organization (WHO), human life expectancy has improved, and most people are likely to live more than 60 years [8]. Older adults are more vulnerable to infectious illnesses, disorders, and hospitalization [9]. According to an estimation made in 2008, the elderly population across the globe was approximately 506 million, which will further be increasing to around 1.3 billion by the year 2040 [10]. Technologies based on wireless sensors provide facilitation to patients and elderly populations specifically—i.e., those who require constant monitoring [11]. The deployment of medical sensor networks in healthcare applications has drastically improved the healthcare sector in the 21st century [12,13,14]. Humans who serve as actors in healthcare infrastructures, such as patients, doctors, staff, etc., have been involved in such wireless networks directly in a limited manner to increase the portability of gadgets and achieve higher transfer rates, along with keeping communication and other beneficiaries secure [15]. Small medical sensors are kept in close proximity to the patient and rarely connected with the body [16,17]. Their service is to monitor changes in the physiological state of the patient promptly. These sensors observe patient vitals and usually communicate them through a gateway to some distant remote site, for detection of possible change [18]. During this process, human intercession is decreased to a minimum level [19]. A specialist can utilize these readings to append an expert appraisal of the patient’s well-being. The most common vital signs include heart rate, temperature, blood pressure, body movement, pulse-oximetry, etc. [20,21]. Such a critical observation and fine-grain attention to detail benefit patients while also making a thorough health history [22].

Although the Internet of things comes with many opportunities, there are many challenges [23,24,25]. Like many other IoT challenges, security has been given a fair amount of attention in the past [26]. This survey extensively elaborates on authentication approaches (both user and device authentication) being utilized and developed for the IoMT specifically.

The rest of the article is structured as follows. Section 2 will discuss the key concepts related to the Internet of things and state-of-the-art healthcare systems. The literature review, Section 3, discusses various approaches related to the authentication process being used at multiple levels, including modern networks such as cloud, fog, and edge. A detailed comparative analysis of existing methods, for cloud, fog, and edge, is explained in the form of tables. Key findings of this study, related to authentication in IoT-enabled health care systems are discussed in Section 4, which is followed up with the conclusion. The list of acronyms used in the paper is listed in Table 1.

## 2. Fundamentals of Internet of Things-Enabled Healthcare Systems

To the disbelief of critics, the IoT had a revolutionary experience in the last decade [27], whereby many ventures have taken place to revitalize hardware and software standards to overcome associated challenges. With many big companies, including Amazon, Microsoft, Intel, Cisco, etc. [28,29,30], and the research community investing efforts and resources into the field, standards of collaborating systems are being created while reducing human interventions, yet achieving quality lifestyles [31].

### 2.1. Internet of Things

The IoT [32], being a network of things, comes with software standards and a variety of electronic sensing devices to collectively address many real-world issues [33]. The “things” in the phrase IoT can be as simple as small home appliances, like ovens or refrigerators, or may be something as complex as a heavy production plant deployed in an industry [34]. According to an estimate presented by Cisco [35], the number of devices connected to the IoT will reach up to 500 billion by 2030. As per a report by IDC USA [36], $1.3 trillion is expected to be globally spent on IoT with projected revenue of $594 billion in the year 2022. With such a promising future, IoT is expanding to conquer many sectors, including smart homes [37,38], smart cities [39,40,41], industries [42,43,44], agriculture [45,46,47], Healthcare [48,49], transportation [50,51,52], and various other sectors [53,54,55,56,57,58].

### 2.2. Internet of Things-Enabled Healthcare Systems

With the exploration of prospective ventures in IoT, its applications in healthcare have shown steady growth over the past several years [59]. The IoMT (also referred to as the medical Internet of things) has emerged, attracting many studies proposing architectures [60], utilizing sensory gadgets [61], securing framework, and management of the things over an IoT deployed in a medical infrastructure [2]. Even though the scope of IoMT cannot be limited, as mentioned in many articles, IoMT encompasses the following broad areas.

- Control over medication and equipment: Although not restricting the scope within a healthcare infrastructure, the IoT empowers the management and control over medication [62] and medical sensory equipment [63]. Continuous as well as real-time monitoring [64] and control of production units [65] can help such automated industries keep up with the challenges faced while meeting end users’ expectations.
- Health data management: It is a fact that in healthcare environmental spaces, such as hospitals, record generation takes place continuously. Though digital systems that help control healthcare infrastructure have been used for a couple of decades, many advancements, including the IoT, can further benefit mankind tremendously [66]. In an IoT-enabled infrastructure, the following tasks are given importance: management of data of patients [67], emergency management [68,69], management of inventory [70,71,72], resource scheduling [73], error prevention [74,75], etc.
- Medical administration and telemedicine: Administration of medical practice and consultation specifically had been limited within hospitals ensuring the physical presence of patients. Covid-19 has made us learn many lessons, including preparedness and remote treatments. Fortunately, with IoT, remote consultation is efficient [76,77,78], and sensory devices play an important role in the recognition of vitals and symptoms [79]. Over the last two years, many e-health systems have been launched to provide proper care to patients across the globe [80,81].

### 2.3. Security Risks and Attacks

**Figure 1:**
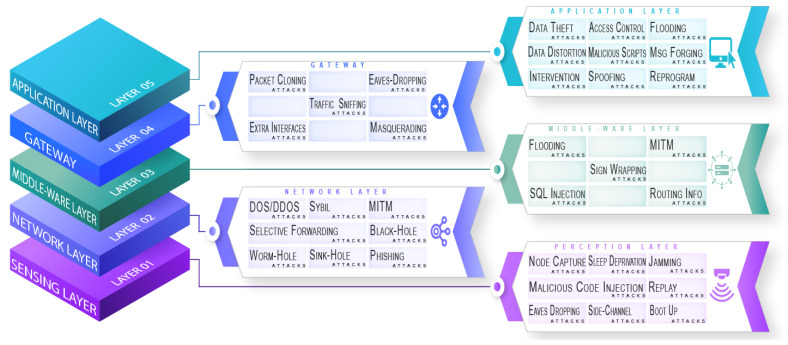
Taxonomy of attacks and threats attempted on IoT [83,84,85].

Security and privacy concerns emerge with every new technological discovery [82]. The IoT brings a paradigm for the interaction of digital devices that have sensory and communicative strengths. Figure 1 provides layers-based taxonomy of possible security attacks in an IoT environment, adopted from [83,84,85].

## 3. Literature Review

Ensuring the security of all the stakeholders in an IoMT environment has been a challenge that has gotten a considerable amount of attention [86]. While performing the review of existing literature, an extensive search was carried out to find articles (both journal articles and conference proceedings) that propose authentication techniques in the context of the IoMT. While skimming such articles, it was observed that the literature could be categorized with respect to the deployment of authentication techniques, such as on the level of cloud, fog, or edge. Moreover, it was also observed that some techniques were generic for both user and device authentication whereas others were specific to each of them. While filtering based on the initial understanding of articles grasped from the title, abstract, and conclusion of the paper, only those techniques were kept that provided insight regarding the approach, testing, and analysis. The following subsections provide a brief description of the working and testing of all such approaches.

### 3.1. Cloud-Based Authentication Frameworks for Patient Monitoring

With the increased usage of the Internet and associated infrastructures, cloud computing offered its three specific models, i.e., SaaS, PasS, and IaaS [87]. Software as a service (SaaS) supplies customers with the infrastructure for running applications. The client accesses the software from other device applications, including a web browser. Platform as a service (PaaS) provides clients with a cloud storage infrastructure. Infrastructure as a service (IaaS) offers clients services, such as configuration processing, networks, servers, and other computing tools [87]. Table 2 briefly compares cloud-based authentication techniques.

As per [93] IBM and active health management, a subsidiary of Aetna (a leading American healthcare provider), based on cloud computing architecture, developed a “Collaborative Care Solution” in 2010. The solution aims to provide convenient access to a wide variety of information from various sources, such as electronic medical records (EMRs), claims, prescriptions, and laboratory data, for medical and healthcare professionals.

**Figure 2:**
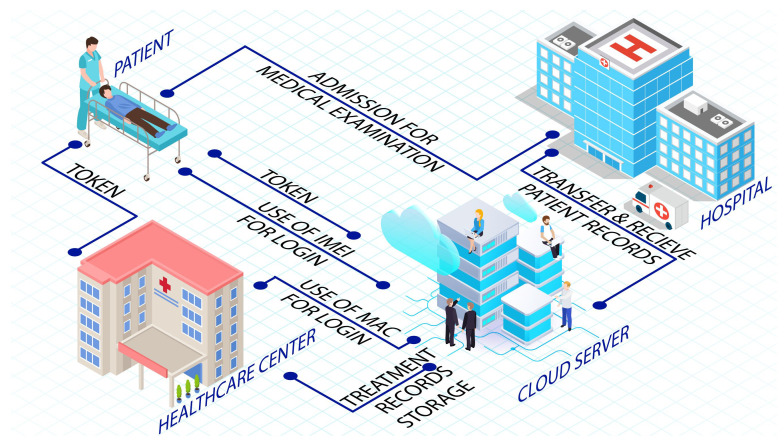
Healthcare authorization model based on cloud authentication [87].

The authors in [87] proposed a cloud-based authentication and authorization scheme using cryptographic techniques as shown in Figure 2. This scheme mitigates medical resource misuse; a patient gets logged in to a public cloud through a mobile device using its credentials (username and password). However, the scheme is kept without a password and identity table being saved in a database. Hence, against attacks such as offline password guessing attacks, replay attacks, impersonation attacks, man-in-middle attacks, and insider attacks, the scheme provides a secure defense. In the proposed scheme, all patients and the hospital administrator are restricted from getting signed in with the public cloud. As a result, a personal attribute is utilized to generate a session key specifically for the user being logged in, such as a patient or hospital administrator. This session key is utilized by patients to get access to all the possible facilities being offered via the cloud. Usually, a patient has to be admitted and provide examinations to receive healthcare services. The healthcare staff then uploads the content with the patient’s healthcare information while acquiring the patient’s approval. Once such information is available on the cloud, any registered user can utilize it for any authorized task. During this process, the patient also permits the use of information through tokens. After successful authentication, a mobile application is used by the patient to generate its authorization token. The patient also has to authorize the hospital; later the authenticity of the patent is validated by the administrator. Once approved by the administrator, the tokens are sent to the public cloud. The next step is to check the token available in the public cloud. If the token is found to be correct, only then can the patient’s treatment report can be accessed from the hospital, and the doctor can supply the patient with the relevant advice. It was reported that the communication cost of the proposed scheme is 7168 bits, whereas the current system requires 0.7168 ms for the data transfer of 10 Mbps bandwidth network infrastructure.

In times of critical sensitivity and decision making, users face difficulty accessing authentic information that is kept encrypted and digitally signed, even after proving its authenticity. Moreover, if an adversary hijacks the IMEI of a patient’s cell phone, it can clone to its own unsafe device and can get access to the network. Thus the scheme is vulnerable to known-key attacks and theft attacks. A user authentication scheme for cloud computing environments using different techniques (i.e., HE-RSA algorithm, AES-192 or AES-256 algorithm, and zero knowledge proof (ZKP) Diffie–Hellman) is proposed in [88]. In this framework, the authors have introduced many agents and a cloud-based SaaS program to validate the authentication mechanism for unregistered devices. In their proposed model, the following entities were used:

#### 3.1.1. Client-Based User Authentication Agent (CUA)

Traditionally, before getting access to a cloud server, a CUA refers to an application placed at the end of the user, or the user’s Internet browser, which is used to validate the user’s identity. In this sort of authentication process, the user’s device gets registered to the website of such a service provider. An extension of the Internet browsers gets downloaded along with a unique encrypted code that provides access to the user. An alternative password would encrypt the unique code the user has selected by using the algorithm AES-192 or AES-256. Thus, to get access, the end user performs decryption of the code and setting up of the extension.

#### 3.1.2. Modified Diffie–Hellman Agent (MDHA)

**Figure 3:**
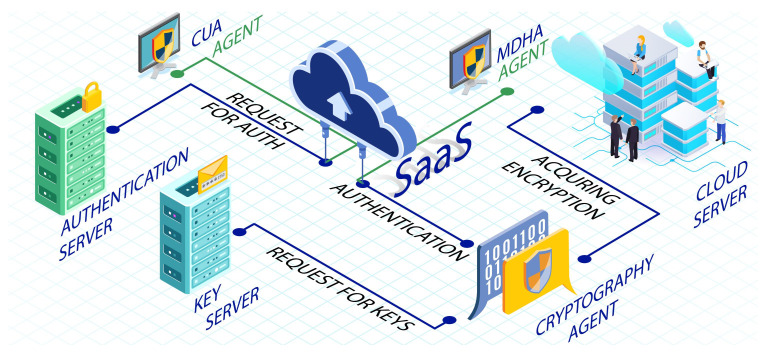
An architecture proposed in [88] for user authentication based on agents and SAAS model.

To improve the reliability rate of unregistered applications in user authentication, MDHA is implemented using zero knowledge proof (ZKP). In this technique, the users will be given temporary permission to access it from the unregistered device. After an extensive analysis of the framework, we have noticed that two separate servers for strong authentication and cryptography are used, which is a resource wastage framework. Therefore, from main servers, it can also be managed and might increase the overall cost and security like mitigating eavesdropping and DOS attacks. The complete scenario of [88] is shown in Figure 3.

**Figure 4:**
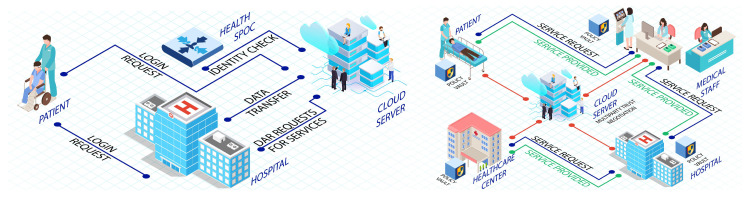
Privacy- and security-enhanced e-health framework [89] and SOTER: A trust discovery framework [94].

The authors in [89] have proposed an enhanced security model with the functionality of authentication and authorization functionality while discovering the new multi-authority encryption-based attribute (MA-EBA) technique to protect healthcare against attacks by unauthorized users as illustrated in Figure 4. The method improves device scalability and allows the user to attain fine-grained access. In the proposed model, the user provides their identity, password, and personal biometric information in the first phase. The admission department generates a request for a service to obtain a request from the database and cloud servers. Upon request by the service administrator, the health requester (DAR) accesses the patient’s stored personal health information. Only DAR can decode personal health information (PHI) after patient satisfaction and access policy definition. When seeking entry to PHI, the single point of contact (SPoC) tests whether the user has been granted access to fetch records from the database. Hence, access to the service becomes the key aspect; thus in a successful scenario, the administrator can access patients’ information. Therefore, the information, PHI, is sent while utilizing the MA-EBA framework, which has been presented in Figure 3. However, if an adversary gets too close to the patient, it can acquire its login information and cause damage to the system. Therefore, the proposed scheme is vulnerable to hijacking attacks.

A framework is proposed in [94] for the authentication of healthcare devices using the Soter platform. This framework, also presented in Figure 4, has been reported to have many state-of-the-art features, including multiparty trust negotiation, to maintain trust amongst connected devices. Moreover, the scheme promotes the use of virtual federations (VFs), and trust circles (TCs) to attain a more robust experience having a customized and dynamic access control policy (DACP) intact. It also utilizes trust negotiation (TN), which is a very innovative approach to managing trust between two individuals having no information about each other.

The proposed framework notably simplifies the process of trust discovery. The transfer of credentials back and forth is decreased in the proposed scheme. This helps reduce communication costs while enhancing the privacy of stakeholders who are actors in the process of negotiations. In addition, it encouraged people to adapt their policies, which regulate the disclosure of their resources or credentials. The IoMT’s credibility rating depends upon trust in the services it provides. The framework describes three trust levels in their research article, fully trusted, partially trusted, and nontrusted. The associated devices’ authentication certificates are deposited in the credential vault (CV). The trust assessment module is carefully designed to keep track of trust and help share a stakeholder credential depending upon the trustworthiness of the one seeking it. It has been reported that if the degree of reliability is higher, very few requirements would be needed during the process. In the proposed model, a SEMTN trust communication technique is also introduced. It is a multiparty trust communication mechanism that enables the system to generate and manage trust between parties through the incremental implementation and dissemination of signed credentials. It also uses a negotiating approach to look for effective access control policy (ACP)-based negotiating. In the proposed architecture, the policy evaluation module decides which certificates are supposed to be sought by other peers within the IoMT system. Furthermore, it also determines which certificates can be sought from other peers and can be revealed by using the SEMTN technique.

**Figure 5:**
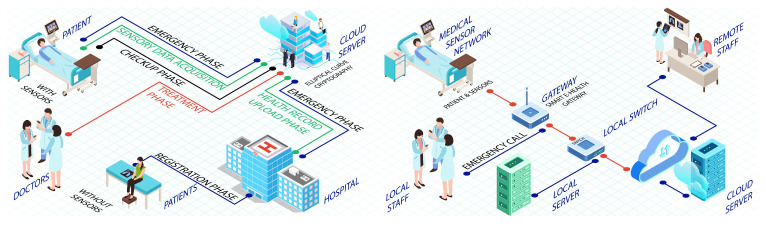
CSEF: Cloud-based secure and efficient framework [90] and An end-to-end security architecture for healthcare IoT [95].

An elliptic curve cryptographic-based framework for smart medical systems is proposed in [90]. The architecture works on wireless sensor network (WSN) technology, wherein the doctor provides a patient with online healthcare services via a cloud-oriented application program on a mobile device. It is reported that for such scenarios, security and privacy are the main issues for the users of cloud-based smart medical systems. Therefore, they designed an architecture to ensure security and privacy by using lightweight cryptographic key generation elliptic curve cryptographic for the proposed smart medical system. The four entities—patient, doctor, cloud server, and health care centre—are passed from their proposed scheme. These entities are processed in six phases: registration, smart medical system, uploading patient health records, treatment and checkup records, and a unique emergency phase, as shown in Figure 5.

In the proposed framework, the patient gets registered with the healthcare centre, which then controls the user’s session key by using the cloud. The healthcare centre sends patients’ medical records to the cloud. Moreover, even at the patient’s request, data gathered by the medical sensors is uploaded. The doctors access such records through their mobile devices and can provide expert opinions and advice remotely. Afterward, using the same method, patients can attain access to medical observations which a doctor and healthcare centre generate. When the patient has some emergency or issues with their pulse rate, respiratory system, heartbeat, etc., the intelligent body sensor informs the cloud. At the same time, the cloud reports onward to the healthcare centre. The said architecture is difficult to implement because the elliptic curve cryptographic (ECC) method consists of an arithmetic encryption function, digital signature function, and verification function. ECC software implementation needs moderate speed whereas hardware implementation consumes more energy, and scalar multiplication is time-consuming in ECC-based schemes. Zp (a group under multiplication modulo of prime number p), which comprises integers modulo having a large prime number p, is considered a challenge for ECC-based logarithms in finite fields. Similar to field sieve, this problem has some subexponential time solutions. It is known that, given sufficient processing strength, subexponential time solutions can be broken down by adversaries in a time of a few months. Thus, in this case, it cannot be considered practical. However, ECC has potentially fallen in the implementation footprint due to the smaller key length alone.

When considering wireless sensor networks (WSNs), ref. [91] thoroughly examined three-factor authentication techniques, and based on the examined flaws of [96], an improved three-factor authentication approach was explicitly proposed for monitoring patients remotely by using WSNs. The revocation and re-registration phase was improved, which was confirmed by BAN logic in the form of a successful mutual authentication process. The AVISPA tool called “Automated Validation of Internet Security Protocols and Applications” was utilized to perform security analysis, where comparison was built with existing four other techniques including [96]. Security comparison was drawn, keeping 17 different security threats and the schemes were also compared for their respective computation and communication cost. It was reported for the proposed scheme that through simulation, resilience was observed for active and passive attacks. Whereas the informal security comparison assured that the mandatory security attributes were available in the proposed scheme that ensures efficient yet secured remote healthcare monitoring of a patient.

With the help of fuzzy logic, the authors of [97] have proposed an approach for the management of trust to authenticate devices and counter sybil attacks in IoMT, which leads to the generation of many fake nodes and imitating a real node to attain malicious objectives. By using fuzzy logic and associated filters, this novel technique calculates the trust score of nodes in an IoMT network based on submeasures like integrity, receptivity, and compatibility. By using simulations, a thorough comparison of energy, accuracy, packet delay ratio, trust computation, and quality of experience (QoE) is built with three other trust-management schemes, such as RobustTrust, SGSQoT, and GroupTrust. The evaluation results declare the proposed scheme significantly better than the rest; however, the authors comment that the overhead cost of various aspects related to servers and the time taken for the delivery of packets can further be reduced to enhance the performance.

In [92], the authors have proposed a lightweight user authentication scheme. The proposed scheme helps monitor patients by using an insecure IoT-based framework. The scheme validates legitimate users to access patient data stored on a cloud server from a remote location. Hash and XOR functions have been utilized by the proposed scheme, which is then analyzed to be less costly with respect to computation complexity in comparison to five other schemes. The proposed scheme is implemented in different phases with assigned roles. First, the registrations center sets the parameters in offline mode, and then the medical professionals and patients get registered to the gateway node, respectively. Additionally, various entities involved, such as the gateway node, sensor node, and user, mutually authenticate each other. A random key for the session is generated after authentication is found to be successful in comparison to the saved information. Furthermore, authenticated users are also provided with the facility to change their passwords, hence replacing previously stored information.

Similar to [91], AVISPA is also used to validate the proposed scheme of [92] for robustness against possible threats and evaluate formal security standards. Comparing the proposed scheme’s computational cost with existing schemes confirms the proposed technique’s efficiency.

In [98], the authors propose a strategy addressing the privacy and security concerns of centralized medical record storage on the cloud-based system being generated by IoMT. It is reported that the proposed scheme is structured on blockchains and also on interplanetary file systems (IPFS) technology. The primary purpose here is to provide a distributed structure for the storage of records. It also ensures the authentication of different devices such as clinical gadgets. The use of these technologies helps in addressing security concerns associated with IoMT-enabled healthcare. Blockchain-based architecture assures that the system is decentralized and the patient and their medical devices are presented with a registration-based security model. A consortium blockchain is built and executed to ensure access control. However, a few issues have been recognized, namely that sustaining a distributed cluster by using IPFS and establishing a distributed cluster system, requires more processing time.

### 3.2. Fog-Based Authentication Frameworks for Patient Monitoring

Cisco Systems introduced the term fog computing in 2014. The term was coined because of its association with closeness to the earth. Similarly, a layer was created between edge and cloud, enabling software or services to be corrected and improvised [99].

Fog is a modern architecture, having a sense of processing, storage, and control that takes the resources closer to end users. The decentralization of resources at the edge of the network is done. Computation and control, both closer to the sensors, make the fog idea a more robust alternative to the cloud [100]. In addition, it encourages the versatility of users while keeping resources heterogeneous. It also acts as an interface, provides data analysis, and meets the requirement of low latency and hence has been utilized in distributed environments [101]. To meet the challenges of today’s healthcare, fog computing is considered a critical competitive platform that assists the cloud in reducing delays, jitters and transmission costs and enhancing throughput [102]. A comparison of few Fog-based techniques are mentioned in Table 3.

The datagram transport layer security was worked on and enhanced by [95]. This security layer works between the two vital entities of fog-based architecture, i.e., gateway and end users. It was suggested that certificates were not required during the initiation of the session. The security was then analyzed of the proposed end-to-end security scheme by using the complete prototype healthcare method and keeping the hardware performance constant. The proposed architecture consisted of medical sensor network (MSN), Gateway, a powerful computer system, and a web interface (application program). However, each participant calculates their key each time, creating a privileged insider attack. Moreover, identity information no longer forms the entire public key. The proposed secure and efficient authentication (SEA) architecture utilizes distributed gateways to safely and effectively perform the authentication and authorization processes on behalf of medical devices. The MSN captures biomedical and surrounding signals from the body/room to monitor and diagnose medical conditions in the proposed framework. The signals are then forwarded through wired or wireless communication protocols to the smart e-health gateway. With the help of communication protocols, the gateway works as a link between the MSN and the Internet. The backend infrastructure consists of a network switch, a cloud computing platform, and a central database (DB) for healthcare, which is shown in Figure 5.

**Figure 6:**
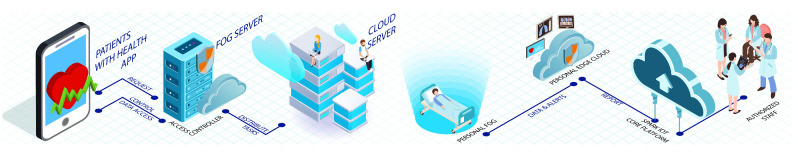
An access control model for the IoMT proposed in [103] and framework of healthcare application [104].

The fog-based access control model is proposed by [103], which protects the performance of cloud/fog-based IoMT. A fine-grained access management framework has been considered for the framework, which is shown in Figure 6. In this scheme, a cloud-based approach is applied by using an additional layer of fog servers. An access control environment is created by using this fog layer, which provides personalized access to the end user. Despite many similarities in storage and application between cloud and fog computing, fog computing differentiates from cloud computing in geographical distributions because it mixes centralized and distributed computing. The proposed approach’s fundamental objective is to minimize the danger caused by using extra assortments by cloud-based applications. It establishes the grounds for essential issues in consent approval, which by default currently is configured by the operating system. For the most part, the standard contemporary settings are reported to be coarse-grained, and for most clients, it is difficult to change such settings. In the proposed framework, both permanent and temporary data are used to preserve privacy.

Access controller is the leading participant on the other peer called the fog server of the proposed scheme. The access controller subelements include register, repository metadata, and repository criterion. A register in access controller shall communicate with different applications. Repository metadata is responsible for compiling information, but it is not considered a shared space. The repository criterion is storing a specific privacy level setting. However, many attributes affect the execution time in such a scenario. If the number of attributes is increased, the performance of the model will degrade. The proposed scheme is also found vulnerable to several threats, such as impersonation and parallel session key attacks. It was also observed that the scheme lacks mutual authentication.

A proposal in [104] demonstrated that IoT architecture uses edge computing. This architecture uses extensive nodes for data transmission, which can disturb the application software models and create confusion. For this purpose, they prefer fog computing for healthcare industries to facilitate the application software models and enhance monitoring functionalities. They further stated that evolution is performed by utilizing smart devices by the patient by using a fog node for sharing sensitive personal information securely with physicians. The specified physician supervises their health situation and proposes preventive measures to the administrators in an emergency. The SparkIoT Platform prototype comprises three groups (i) wearable devices (which act on a personal level), (ii) a mobile application (this ensures access to a private edge cloud), and (iii) the Spark IoT platform core (the platform is deployed on the cloud). The first group consists of smart sensors attached to the patient. The patient’s health data in encrypted form and alerts are stored in the storage of wearable devices. The mobile application is installed on the patient’s cell phone and connected to the wearable devices to receive and store the alerts and traces. The mobile application manages wearable devices, patient body sensors, battery storage, alerts, and algorithm parameters. The third group Spark IoT platform core, provides secure user authentication, personal health assistance, access to the medical staff, and maintenance of patients’ electronic health records (EHR). The proposed framework is insecure because all the data and alerts are stored on the mobile device; the attacker can steal it. Figure 6 shows the working of the proposed Spark IoT platform.

It was observed that radio frequency identification authentication is utilized on many occasions for the IoMT to identify end nodes and users. In order to avoid tag collisions of RFIDs, a scheme is proposed by [105] using aloha. The scheme uses the dynamic framed slotted aloha, which is an anticollision protocol [106]. The three components of the proposed model include numerous batch tags, readers, and backend servers. Every product carried by the patient is attached with an RFID tag, and wireless channels are used to interact with them. An RFID batch authentication technique is presented to minimize tag costs and increase tag-recognition efficiency. Furthermore, a linear homogeneous equation is utilized in the RFID batch authentication system. The proposed scheme is a two-phase technique that includes the startup phase (registration) and authentication phase. To decrease the computational cost of batch authentication the properties of homogeneous linear equations are used. By sing Vivado, environment timing and behavioral simulation based on FPGA have been carried out in comparison to other super-lightweight authentication techniques. The proposed technique is found to be more secure and accurate, having less computational cost. However, comparisons on real-world scenarios in the field of medical health have not been made.

### 3.3. Edge-Based Authentication Frameworks for Patient Monitoring

With the takeover of technology in hospitals for medical applications, considerable efforts of the research and industrial community are being put into an edge-computing provision in health infrastructures. One of the critical challenges of IoMT systems focused on edge computing involves maintaining the power of medical equipment and raising the lifetime of the healthcare system [107]. Wireless body area networks are equipped with different sensory modules and gadgets. These sensory gadgets are placed around, in, or on the human body [108]. All such devices act as nodes and are usually linked through wireless communication technologies. WBANs may include wearable and implantable biosensors for remote observations, medical assistance, and other remote services [108]. Table 4 elaborates key features, attacks overcome and limitations of some of the recent work in Fog-based authentication.

**Figure 7:**
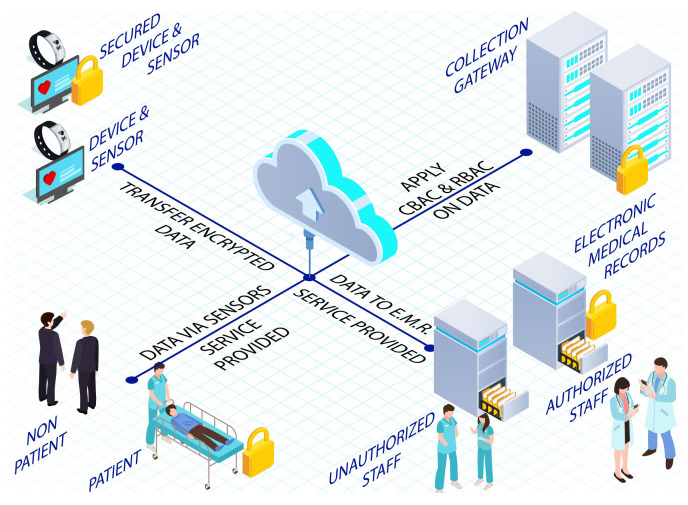
Service-oriented security framework in the IoT, proposed in [109].

As discussed in [109], high-speed ICT tools are usually installed for remote patient monitoring and supervision. It is noted that such an environment usually lacks security, which can lead to many issues for all the participants of such an environment. Therefore, they proposed a framework that resists all known threats inside the IoT. In the proposed architecture, as illustrated in Figure 7, first of all, data from the patients are verified by using device authentication (DA) and transferred after securing the channel (SC) and applying data encryption/decryption (DED). Access control models are applied at the gateway level, which includes contextual-based access control and role-based access control. DED and SC can also be used for additional security. Data is then transmitted to the hospital’s electronic medical record (EMR), where user authentication (UA) is used with DED, SC, CBAC, and RBAC. After the authentication process, the services will be provided to a patient remotely. Their result shows that they have proposed a secure mechanism that ensures the participants’ confidentiality, authorization, and privacy. With an in-depth study of the framework, it was observed that the proposed framework is not fast and secure. A GPU is required to enhance performance, while simple encryption/decryption cannot guarantee security. A random and robust key is necessary for the mutual transmission of data among all the participants.

**Figure 8:**
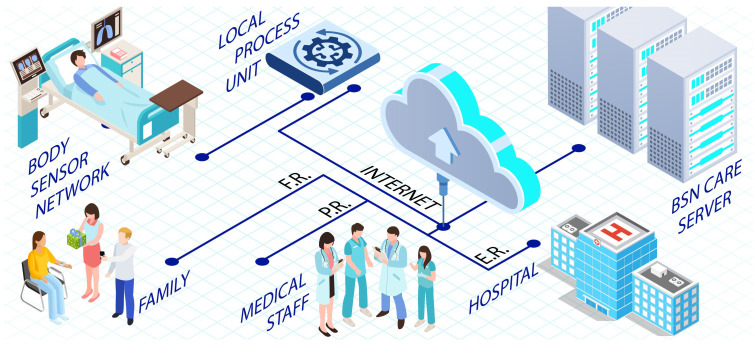
BSN-Care: IoT-based healthcare system using body sensor network as discussed in [110].

A study in [110] focused on the significant issue of security in the healthcare system and proposed a secure architecture by using the lightweight anonymous authentication protocol, namely BSN-Care. BSN architecture consists of wearable and implantable sensors. The physiological parameters of the patients are collected and transmitted to a local processing unit (LPU) such as PDA or smartphone. A central server termed the BSN-Care server controls the data flow between the nodes and LPU. Databases store medical records received from LPU, which are then analyzed for possible abnormalities. The degree of irregularities would determine if the family members and doctors are to be contacted or not. In case of extreme abnormalities, services of any emergency units in the close vicinity of the patient will be acquired. Such actions will be reflected in the action table through boolean variables as family response (FR), physician response (PR), and emergency response (ER). There are two phases within the authentication protocol. In the first of registration, the central BSN-Care server issues IDs to all the LPUs connected through a secured means. During the authentication phase, the LPU and BSN-Care servers mutually authenticate each other. Data transmission takes place after this phase. It was observed that if an adversary somehow gets access to an already authenticated LPU, it can quickly figure out the identity, masquerading and impersonating the whole system. The working of the proposed architecture is graphically represented in Figure 8.

Therefore, the scheme fails to provide secure services. Similarly, while looking into the first message transmission, it is noted that the message is sent publicly over the network channel, in which an imposter can very easily attempt a replay attack. Also, the attacker can discover the identity. It can easily identify users’ personal sensitive information, like location and the session start time. Thus, it can easily trace a legitimate user by launching a traceability attack over it.

**Figure 9:**
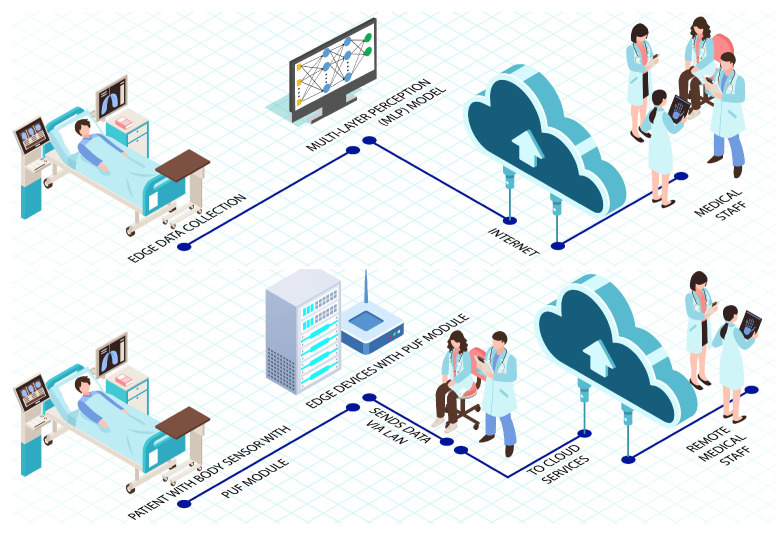
Framework of multilayer security scheme for implantable medical devices [111] and PUF-based energy-efficient authentication IoMT [114].

Another study in [111] suggested a novel electrocardiogram authentication scheme using Legendre approximation integrated with the IoMT-enabled multi-layer security approach as shown in Figure 9. To provide network, data, and application-level security, they utilized wireless implantable medical devices (IMDs). In this scheme, all the QRS coefficients of legal doctors are stored inside the IMD of the patient to give authorization. Different doctors are given different privileges and permissions. Based on user IDs, complex and adaptive access control can be implemented in IMDs. An ECG machine is used as biometrical signal input in the application layer. Unique identities were assigned to patients to utilize the ECG signals. At the network layer, coefficients and unique identities become passed via a direct sequence spread spectrum method of encryption that guarantees the coefficients of authorized people. MLP classifier model works on the data link layer where the ECG signal is evaluated on a temporal basis. Another purpose of the MLP classifier is to protect the accuracy and completeness of the information.

However, no one accepts it as a standardization model, as it cannot adopt any changes in the biometric phase of a collection of samples. It also influences the environmental and mental conditions of a patient.

A study conducted in [114] introduces a computer authentication protocol used to authenticate network devices. The authentication process is performed without data being stored in memory. In the PMsec method, as shown in Figure 9, each device would incorporate a PUF module. All the sensors and devices in the IoMT get their unique identities from this module. The protocol’s initialization happens any time a new device establishes a link with the network. The server utilizes the PUF module to enroll different keys throughout the process. A REQUEST input R1 is transferred to the said module. Consequently, a RESPONSE is given to the PUF module at the end devices for R2 response. The second response (R2) is again sent to the said module. Furthermore, a third response (R3) is generated where the hash of the same is also calculated as X = h (R3). The hash X and CHALLENGE input C1 are contained in the database. The device inputs a challenge message toward the node, which produces a hash value called X/. Hash values X/ are matched with the already stored value X for authentication of the device. However, device information is not stored explicitly in the cloud log, which provides an extra layer of security to the system.

In [112], keeping the context of IoT-enabled healthcare systems, the authors have presented a lightweight authentication technique for WBAN, which is based upon two factors. The proposed scheme authenticates users and devices available over an IoT. The authors utilized ProVerif and OPNET tools to simulate and analyze the proposed scheme. Results have shown that key compromise impersonation attack and known session-specific temporary information attack have been countered while providing forward secrecy. Moreover, the real-or-random model, also called ROR model, is used to perform a formal analysis of the security of the proposed scheme.

A device authentication technique is proposed in [113] by using the SVM classifier model while keeping [115] as their base model. The sensors and gateway communications in an IoMT architecture are secured by machine learning in trust management strategies and authentication techniques. The tests show that the proposed schemes work well with various IoT-based medical frameworks with a lower computing cost than the physical unclonable functions (PUF) protocol. The proposed approach is found to be secure, efficient, and resource friendly.

To ensure the privacy of credentials in a scenario in which the session secrets get revealed to an attacker, a device authentication scheme for WBAN’s is proposed in [116]. While avoiding the management of public keys in a large number, the CK-adversary model is utilized to provide strong security of credentials. By using Java pairing-based cryptography library (JPBC), the session keys are evaluated for communication, computational, and storage costs. The proposed scheme is efficient and more secure, suitable for telehealth applications.

In [117], the authors have proposed an authentication scheme claimed to be lightweight, namely slight. The technique is used for smart healthcare services to address security challenges while providing a lightweight architecture. This architecture is built to enable end users to receive medical advice from experts. Keeping three main entities, i.e., doctor, medical server, and sensor or patient, the system includes four phases: registration, authentication, transfer of rights, and update of password phase. Security analysis concerning resilience against security threats of the proposed scheme is performed and formally analyzed by using the Scyther tool.

If an adversary knows the IDs or secret keys, it still cannot retrieve the session key, and thus the proposed techniques are found to be suitable for forward secrecy. During the authentication process in slight, timestamps are embedded with messages to calculate their freshness. Moreover, a technique involving the use of random numbers is utilized to counter the denial of service attack and bound any adversary from sending repetitive messages. It is reported in [117] that the time complexity of slight is 0.0076 ms, which is very low. It has been analyzed that the communication overhead is also quite acceptable compared to similar frameworks. These two aspects make the proposed model suitable for any IoT-based solution with limited computational power at various sensors and devices.

### 3.4. Comparison with Other Surveys

Over a few years, many thorough surveys have been conducted in the broad area of IoMT while keeping various categorization strategies in discussing the existing literature. Table 5 provides a brief description of the taxonomies adopted by the surveys conducted for IoT-enabled Healthcare systems. Moreover, other security analysis details are also listed in the referred table.

## 4. Key Findings

The current state of the world, especially after encountering a deadly virus, i.e., covid, implores us to bring coherence in our efforts to provide better health services specifically while using state-of-the-art modern practices. While exploring authentication practices in the Internet of things enabled healthcare systems during our survey, we witnessed a sufficient amount of effort being delivered into the field. However, this section will attempt to raise questions and highlight pitfalls that may be addressed to pave the way forward in providing quality health services through modern communication technologies.

### 4.1. Lessons Learned

With the review of existing literature, we can summarize the lessons learned as follows.

Resource constrained strategies: It is known that the IoMT comes with a constrained environment with only limited storage, communication and processing power. More efforts are to be made to shift toward lightweight strategies that will assist not only in the field of healthcare but also in other IoT-based application areas.

Dataset generation: A considerable amount of effort needs to be focused on creating usable datasets for precise approximation during simulations. It was observed that actual datasets were either unavailable or not made public. Given the sensitivity of the healthcare domain, almost every simulation needs to be tested on real datasets and testbeds.

Standardization: Redundancy was found in the creation of frameworks being proposed by various researchers. A more coherent and standardized approach may be adopted, which will save potential and direct them toward efficiency.

Evaluation: Evaluation of security models was noticed to be not uniform in many of the proposed schemes. The use of formal and informal security analysis practices with the help of simulation-based tools needs to be practiced.

Toward usable security: The aspect of providing usability with security or privacy was found to be very rare. More efforts must be made to bring in user-friendly technologies such as augmented reality, virtual reality, etc., for a better user experience. Steps may be directed to keep humans in the loop while providing security.

### 4.2. The Road Ahead

With a detailed discussion of different existing authentication techniques in the IoMT, we conclude this article with open issues in the respective field. Though the survey is structured with respect to levels of the network including cloud-, fog-, and edge-based authentication, however, keeping in view the limitations of the various approaches, a summarized set of prospect open issues are mentioned below.

- Use of cryptographic keys is found to be abundant in security architectures; still, very little work is performed in creating, managing, and moving such keys in resource constraint environments. Moreover, trusted platform module (TPM) or similar hardware-based solutions may be utilized on various levels of IoT to provide secure utilization of keys.
- In the context of the IoT in general, usability and interfacing of its various layers is compelled to be kept very limited. Usable privacy and security with the help of modern UI/UX standards can help make many efficient solutions. It has also been observed that the end user has been neglected during the creation of specialized solutions, creating a gap in usability and utility in security standards.
- End-to-end authentication of users has yet to be explored, keeping IoT infrastructures and limited resource availability in context. Moreover, the perspective of provision of security standards, authentication in specific, has been limited to a certain number of security threats, and many other attacks may also be given importance, such as cloning attacks, node compromise issues, desynchronization attacks, and masquerading problems, etc.
- Authentication techniques may also be revised to provide better security and privacy to different types of end users of the IoT. It has been observed that the process of revamping of security standards, specifically authentication techniques, improves the security of the platform, which is not compromised easily, and the end user stays interested in keeping itself secure and updated. Keeping in view the limitations and strengths of different types of specialized IoTs, end-to-end user authentication may also be improved.

## 5. Conclusions

The IoT has earned its due attention over time. The last few years emphasized the need for technology in almost every sector of life. The IoT has many prospects in applied areas, but healthcare has been one of the most explored fields. This study orbits around authentication techniques being designed for the IoMT. Furthermore, the authentication schemes designed on different levels of the network, such as cloud, fog, and edge, have been analyzed for their contribution and limitations. Similarly, the role of authentication in an IoMT-based environment is studied. The study’s essential findings implore the research community’s attention to focus on providing quality healthcare services through modern technologies.

## Figures and Tables

**Table 1:** Acronyms and their respective full forms.

Acronym	Full Form
AES	Advanced Encryption Standard
CUA	Client-based User Authentication
CBAC	Contextual Based Access Control
CV	Credential Vault
DED	Data Encryption/Decryption
DoS	Denial of Service
DA	Device Authentication
DACP	Dynamic Access Control Policy
ECC	Elliptic Curve Cryptographic
EHR	Electronic Health Record
EMR	Electronic Medical Record
EMR	Electronic Medical Records
HE-RSA	Homomorphic Encryption- Rivest Shamir Adleman
IMDs	Implantable Medical Devices
IaaS	Infrastructure as a Service
IMEI	International Mobile Equipment Identity
IoMT	Internet of Medical Things
IoT	Internet of Things
IPFS	Interplanetary File Systems
JPBC	Java Pairing-Based Cryptography Library
LPU	Local Processing Unit
MSN	Medical Sensor Network
MDHA	Modified Diffie-Hellman Agent
MA-EBA	Multi-Authority Encryption-Based Attribute
PHI	Personal Health Information
PUFs	Physical Unclonable Functions
PaaS	Platform as a Service
QoE	Quality of Experience
RFID	Radio-Frequency Identification authentication
RBAC	Role-based Access Control
SEA	Secured and Efficient Authentication
SPoC	Single Point of Contact
SaaS	Software as a Service
SSDP	Simple Service Discovery Protocol
SEMTN	Stateless Multiparty Trust Negotiation
TC	Trust Circles
TN	Trust Negotiation
TPM	Trusted Platform Module
UA	User Authentication
VF	Virtual Federations
WBAN	Wireless Body Area Network
WMSN	Wireless Medical Sensor Network
WSN	Wireless Sensor Network
WHO	World Health Organization
ZKP	Zero-Knowledge-Proof

**Table 2:** Cloud-based authentication techniques in IoT-enabled healthcare systems.

Study	Technique Used	Attacks Overcome	Main Contributions	Limitations
[87]	Asymmetric cryptography	Offline password guessing, replay, impersonation, man-in-the-middle andinsider attacks	Cryptographic mechanisms, one-way hash function, symmetric encryption and the bit-wise exclusive-or operator are utilized to provide identity authentication and authorization through the cloud. The authors claim that authentication, integrity, privacy, and nonrepudiation issues are resolved.	At times of hurry, information that has been oversecured by encryption and digital signatures can become difficult to access. Moreover, if an adversary gets access to a patient’s mobile or impersonates the IMEI on its cell phone, it can gain access and cause damage.
[88]	Client-based user authentication agent and modified Diffie–Hellman agent	Man-in-the-middleBrute Force Dimming	Scalable and efficient authentication technique is proposed. A cryptography agent is introduced to encrypt data before its storage	Use of multiple servers increase overall computational and communication cost. Data headers for transmission are not tagged and can cause additional overhead cost.
[89]	Multiauthority attribute-based encryption	Man-in-the-middleEavesdroppingDOS	Advance encryption standard is used to make the data secured. It is explored how a single point of contact can assist security e-health	Very limited analysis is performed and the approach may be prone to attacks like shoulder surfing attacks, impersonation attacks, etc.
[90]	ECC and hash function	Man-in-the-middleImpersonationNonrepudiationTraceabilityReplay	HIPAA compliant framework ‘SOTER’ is proposed which is distributed personalized authentication based on MTN. Limitations of Identity Access Control Policies are attempted to be resolved primarily.	Limited Authorization model, having only a few stages. Formal or informal security evaluation lacks.
[91]	Multifactor mutual authentication	Reply attack, DOS, smart card loss attack, password guessing attack, etc.	An improved three-factor authentication approach is proposed specifically for the monitoring of patients remotely using WSNs. AVISPA Tool is utilized to perform formal security analysis	Communication cost is a bit high, but still, extensive evaluation has been performed.
[92]	Hash and XOR functions, lightweight key management	Malicious user attack, replay attack, password guessing attack, insider attack, hidden server attack, spoofing attack	A lightweight user authentication scheme is proposed to validate legitimate users using Hash and XOR functions while minimizing the number of cryptographic computations.	The scheme is lightweight due to the use of computationally constrained functions; however, it lacks security against some attacks such as shoulder surfing, eavesdropping, etc. An extensive security evaluation of various platforms is not provided.

**Table 3:** Fog-based authentication techniques in IoT-enabled healthcare systems.

Study	Technique Used	Attacks Overcome	Main Contributions	Limitations
[95]	ECG-based cryptographic keys and certificate-based datagram transport layer security	EavesdroppingDoSSpoofing	The study explores an efficient end-to-end user authentication scheme assisted by DTLS certificate handshaking. It also stated to provide a session resumption feature with mobility as it builds smart gateways within the network.	Proper computational and communication analysis is performed, whereas formal or informal analysis of the scheme is missing.
[103]	Fog-based security and access-control determination algorithm	SpoofingMan-in-the-middle	This work proposes a fine-grained security, access control mechanism in specific. Reported suitable for various services like data storage, directories, and file management, while providing customized security features	Quantity of tasks is found out to be directly proportional to time complexity. Hence, in a scenario where tasks increase, time complexity will affect.
[104]	A core IoT platform, ‘Spark’	Insider attack	Primarily a framework is proposed comprising of layers to increase the efficiency of data transfer and throughput while providing an additional layer of security and authentication.	Through network simulations transfer of health care data such as ECG is examined. No information regarding security or privacy preservation analysis is provided.
[105]	Dynamic framed slotted aloha and RFID	Tag-tracking attack, replay attack	An RFID batch authentication technique is presented to minimize tag costs and increase tag recognition efficiency. Furthermore, a linear homogeneous equation is utilized, and the scheme has a registration and authentication phase.	Tag anonymity and mutual authentication are provided yet lack formal evaluation of the security. Impersonation attack should have been dealt with.

**Table 4:** Edge-based authentication techniques in IoT-enabled healthcare systems.

Study	Technique Used	Attacks Overcome	Main Contributions	Limitations
[109]	Dynamic adaptability to changing in security needs through access control models	Device masquerade attack, spoofing, denial of service, Reflection attack, Eavesdropping	Service-oriented structure is proposed with the support to dynamic elements of security. These elements continuously change based on medical service providing remote points and are secured by assisted roles and situation-based access controls.	Preliminary comparison and security analysis is performed with no hint of evaluation details. Formal and informal security comparison needs to be performed.
[110]	Body sensor network-based architecture along with use of local processing unit (LPU)	Forgery attack, eavesdropping, False signal attack, Replay attack	Body sensor networks were used to propose an IoT-based healthcare system assisted by OCB to fulfill five security requirements, i.e., mutual authentication, enforcing anonymity of actors, secured localization, resistance to security attacks and data security.	Extensive computational analysis is performed compared to two BSN-based models; however, only security features are listed. The scheme may be prone to threats such as impersonation, lost key, and shoulder surfing attacks.
[111]	Legendre approximation of ECG and multilayer perception neural model	Eavesdropping, replay, and man-in-the-middle attacks	The method of Legendre polynomial extraction is used to propose an ECG authentication technique. Multi-layer perception neural network is also utilized for learning, identification, and authentication by using ECG signals.	The possible errors in the acquisition of ECG signals are not discussed. Security analysis, other than machine learning, should be performed.
[112]	Lightweight hash-chain-based and forward security enabled scheme for WBAN	impersonation attack, guessing attack, user/gateway forgery attack, insider attack, and DOS attack	A two-factor authentication scheme for both users and devices is proposed. ROR model is utilized for formal analysis, whereas, ProVerif is used with OPNET utilized for real-time simulation-based evaluation.	Even though a thorough analysis is performed, it seems like the cost of storage and communication is high for the proposed scheme.
[113]	Machine learning (SVM), pseudo-random binary sequence, trust management	Impersonation attack, denial of service attack, man-in-the-middle attack	Machine learning-enabled IoMT network to provide security, trust management and to achieve efficient authentication. Key agreements and trust values are based on securing the IoT healthcare system	There is no use of cryptographic functions; moreover, the formal and informal security analysis is needed.

**Table 5:** Overview of existing surveys of security in IoT-enabled healthcare systems.

Study	Title	Year	Description	Publisher
[26]	A Review of Security and Privacy in Internet of Medical Things (IoMT)	2019	Classification of Security aspects and protection mechanisms, (Device, Connectivity & Cloud Security), Categorization of Privacy aspects and protection mechanisms, (Private Data, Protection Mechanism, Identification & Anonymity, Data Destruction)	IEEE
[49]	Towards Secure and Intelligent Internet of Health Things: A Survey of Enabling Technologies and Applications	2022	Requirements, Security Challenges, Attacks in IoT based Healthcare Systems, Enabling Technologies for Secured IoHT (Convergence of Blockchain, Machine Learning and IoT), Future Paths and limitations of Existing Solutions	MDPI
[118]	A comprehensive survey of authentication methods in Internet-of-Things and its conjunctions	2022	Classification of IoT Security parameters and objectives, Categorization of Authentication Scheme in IoTs (WSN based, IIOT based, IoMT based, VANET based, RFID based),Future directions	Elsevier
[119]	Security and privacy for the IoMT-enabled healthcare systems: A Survey	2019	Systems, networks, and design challenges for IOMT, security and privacy requirements, existing security schemes, discussion and future directions	IEEE
[120]	A systematic review of IoT in healthcare: Applications, techniques, and trends	2021	A systematic review leading into Comprehensive taxonomy for IoT-based healthcare systems (Sensors, Resource, Communication, Application & Security), Comparison of Analysis techniques and research objectives, Open Issues and Future directions	Elsevier
[121]	IoT Security in Healthcare using AI - A Survey	2021	Security for IoT and its types (Physical and Information), Classification of Security in IoT-Healthcare (IoT Security in Healthcare, AI Security in Healthcare, IoT Security in Healthcare using AI)	IEEE
[122]	Review of security challenges in healthcare internet of things	2021	Discussion about Security Issues in IoMT, Identification of Primary security risks, Risk Analysis and Impact Detection of Primary Security Threats	Springer
[123]	Secure Remote User Authentication Scheme on Health Care, IoT and Cloud Applications	2021	Systematic Review resulting into categorization of Remote User Authentication, Tele-medicine Application, IoT Applications, Cloud and multi-server Applications, Possible Security Requirements and Attacks	Acta Polytechnica Hungarica
[124]	A Survey on Security Threats and Countermeasures in Internet of Medical Things (IoMT)	2022	Architecture of IoMT Edge Network and its Security Objectives (Data Confidentiality, User Integrity, Non-repudiation, Authentication, Authorization, Availability), Categorization of Threats and Attacks, Countermeasures for all such security risks	Wiley
[125]	Systematic Review of Authentication and Authorization Advancement for IoT	2022	Taxonomy of Authentication and Authorization Techniques for Iot (Years-based, Goals-based, Automation-based), Dominant Topologies, Communication types and Perspectives in Authorization and Authentication, Applicability of identified solutions	MDPI

## Data Availability

Not applicable.
